# Supplementary figures and images for: Stillbirth rates, service outcomes and costs of implementing NHS England’s Saving Babies’ Lives care bundle in maternity units in England: A cohort study
Source: PLoS One. 2021 Apr 19;16(4):e0250150. doi: 10.1371/journal.pone.0250150 (PMC8055032; doi:10.1371/journal.pone.0250150)

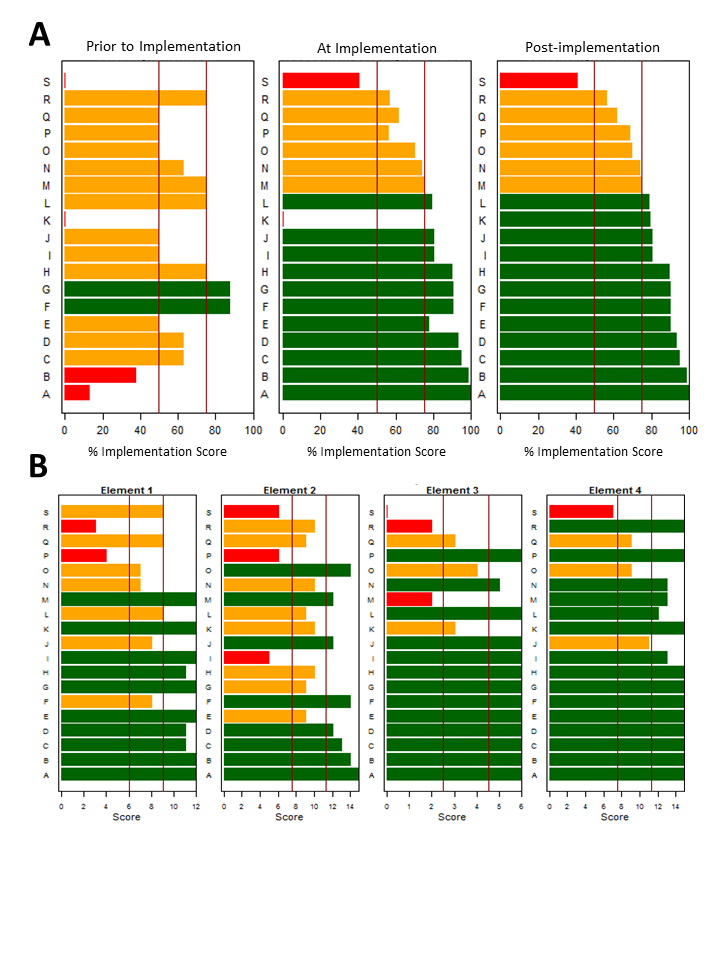

Supplement: S1 Fig — A) Average implementation of all elements at different time frames of the project. B) Level of implementation of each elements at the post-implementation assessment. The total element scores vary due to different number of components in each Element. Element 1: Reducing smoking in pregnancy (4 components), Element 2: Risk assessment and surveillance for fetal growth restriction (5 components), Element 3: Raising awareness of reduced fetal movements (2 components) and Element 4: Promoting effective fetal monitoring in labour (5 components) Red = <50% of element implemented, Orange = 51–75% of element implemented, Green = >75% of element implemented. Republished from Saving Babies’ Lives Project Impact and Results Evaluation (SPiRE): A mixed methodology study under a CC BY license, with permission from the University of Manchester, original copyright 2018. (TIF) [file pone.0250150.s002.tif]

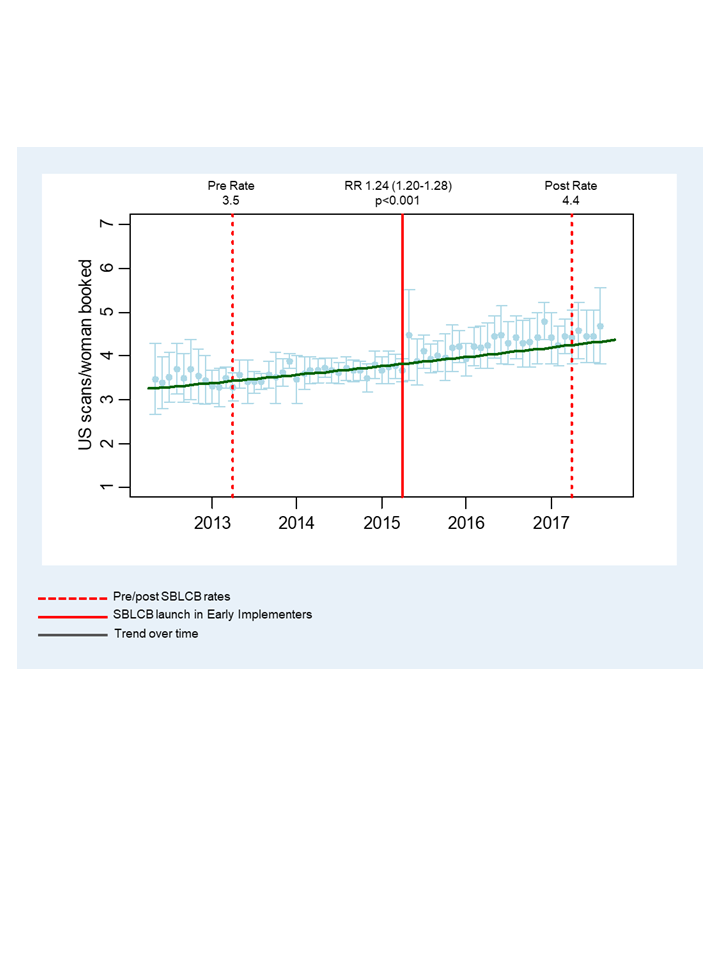

Supplement: S2 Fig — Across participating sites pre and post implementation of the Saving Babies Lives care bundle demonstrating a 24% increase in the number of ultrasound scans performed. Republished from Saving Babies’ Lives Project Impact and Results Evaluation (SPiRE): A mixed methodology study under a CC BY license, with permission from the University of Manchester, original copyright 2018. (TIF) [file pone.0250150.s003.tif]
