# Supplementary material for: Stillbirth rates, service outcomes and costs of implementing NHS England’s Saving Babies’ Lives care bundle in maternity units in England: A cohort study
Source: PLoS One. 2021 Apr 19;16(4):e0250150. doi: 10.1371/journal.pone.0250150 (PMC8055032; doi:10.1371/journal.pone.0250150)
Supplement: S1 Table — (DOCX) [file pone.0250150.s004.docx]

**Table S1.** Sensitivity analysis and alternative costs for health economic analysis

| **Element 1** | **Element 2** | **Element 3** | **Element 4** | **Scans** | **Inductions** | **Deliveries** | |
| --- | --- | --- | --- | --- | --- | --- | --- |
| **Base case** | | | | | | |  |
| **£1,394,713** | **£391,000** | **£66,605** | **£1,798,039** | **£33,765,735** | **£28,945,817** | **£26,754,741** | |
| 9 monitors/  1000 births | 100% of units use GAP software (132) | Leaflet given out once, no additional visits costed | Training costs £60pp; midwives, junior doctors, consultants training | Cost per scan is £52.94 (NHS reference costs 2015/16 - ultrasound scan <20 minutes) | Observed ~20% increase from 26.27 per 100 births to 31.40 per 100 births | Assume that additional elective sections would have been normal deliveries | |
| **Sensitivity 1** | | | | | | |  |
| **£2,465,725** | **£195,500** | **£133,210** | **£2,996,731** | **£27,425,890** | **£14,822,741** | **£53,465,760** | |
| highest number of monitors/  1000 births (17) | 50% of units use GAP software (66) | Leaflet given out twice | Training costs £100pp; midwives, junior doctors, consultants training | Scan cost £43 (NHS tariff costs - ultrasound scan <20 minutes) | 10% increase in inductions | Including increase in elective sections | |
| **Sensitivity 2** | | | | | | |  |
| **£323,701** | **£9,742,300^1^** | **£2,569,296** | **£23,254,579** | **£66,230,335** | **£37,056,853** | **£26,754,741** | |
| Lowest number of monitor/1000 births (1) | Includes staff time to complete training in 100% of units (7.5 hours)* | 5% of births attend once re: RFM (£75.15/visit);one leaflet | Training costs £60pp; also includes staff time (15 hours)* to complete training | Scan cost £103.84 (NHS reference costs - antenatal ultrasound scan) | 25% increase in inductions | As base case | |

*unit cost for staff time (per hour): midwives £44; obstetric consultants £104; junior doctors £40 (PSSRU Unit Costs of Health and Social Care)

^1^Based on mean numbers of staff completing training reported by early adopter sites – 40 midwives and 2 sonographers per 1000 births per year

^2^Based on mean numbers of staff completing training reported by early adopter sites – 40 midwives, 3 obstetric consultants, 3 junior doctors per 1000 births
