# Supplementary material for: Stillbirth rates, service outcomes and costs of implementing NHS England’s Saving Babies’ Lives care bundle in maternity units in England: A cohort study
Source: PLoS One. 2021 Apr 19;16(4):e0250150. doi: 10.1371/journal.pone.0250150 (PMC8055032; doi:10.1371/journal.pone.0250150)
Supplement: S1 File — (PDF) [file pone.0250150.s005.pdf]

Maternity Care Survey

What is this survey about?

You are being invited to take part in a questionnaire being run by the University of Manchester on behalf of NHS England. You have been invited because you have recently received maternity care in a NHS hospital. We would like to know about your experiences of and views towards the antenatal care you received during your pregnancy and labour.

What is my information used for?

Your answers will help us evaluate maternity care provided to women and will then be used by the NHS to improve maternity care across the country. Completing the questionnaire will not affect the care you or your baby receives whilst on the unit.

What do I need to do?

- Firstly, the questionnaire will ask you about your care in pregnancy. Some of the questions will be multiple-choice and some will ask you to give some more details about your thoughts and experiences.
- Secondly, it will ask you to answer some questions about yourself, such as your age and how many children you have.
- The survey should take approximately 15-30 minutes to complete.

Can I withdraw my answers from the study?

Because the questionnaire is completed anonymously (the researchers cannot identify you), once you submit your answers, you will **not** be able to withdraw them. Therefore, by completing this questionnaire you understand that your answers cannot be withdrawn and they will be used in this study. Please do not complete the questionnaire if you are not happy with this.

Will I receive anything for completing this survey?

Once you have completed the questionnaire, you will be given the option to provide your contact details for two reasons:

1) To enter you into a prize draw to win a **£100 Amazon voucher** as a thank you for completing the questionnaire. If you provide your contact details for this reason we will only use your details to contact you if you have won the prize.
2) If you wish to receive a summary of the research findings once the study has ended.

Your contact details will be kept separately from your answers and therefore, the researchers will not be able to identify you from the answers you provide. You do not have to provide your contact details if you do not want to.

Hospital Name																
Today's Date			/			/										
**ID No. (for internal use only)**																

What will happen to the information I supply?

The information you supply will be analysed by the University of Manchester's research team. Once the study has ended we will keep your anonymous answers for 5 years in line with University of Manchester's recommendations. The information you supply may be viewed by responsible individuals from the University of Manchester and regulatory authorities. Any contact details you provide will only be kept until the end of the study and then they will be destroyed. We will not pass them on to any third parties.

Can I get more information about the study?

If you would like more information about your participation in the study, have any questions, or would like a summary of the findings once the study has ended (instead of providing your contact details), please don't hesitate to contact:

Dr Kate Widdows (Project Manager)
Maternal and Fetal Health Research
5th Floor Research, St. Mary's Hospital
Oxford Road
Manchester
M13 9WL
Telephone: (0161) 276 6916
kate.widdows@manchester.ac.uk

Dr Alexander Heazell (Senior Clinical Lecturer)
Maternal and Fetal Health Research
5th Floor Research, St. Mary's Hospital
Oxford Road
Manchester
M13 9WL
Telephone: (0161) 701 0889
alexander.heazell@manchester.ac.uk

You do not have to complete the questionnaire if you do not want to.

Please answer the following questions about your most recent pregnancy and antenatal care you received.

1 In which hospital did you receive your antenatal care and give birth?

		Antenatal care	Give birth
1	Musgrove Park Hospital, Taunton	☐	☐
2	Barnsley Hospital, Barnsley	☐	☐
3	Cumberland Infirmary, Carlisle	☐	☐
4	West Cumberland Hospital, Whitehaven	☐	☐
5	St. Mary's Hospital, Manchester	☐	☐
6	Royal United Hospital, Bath	☐	☐
7	Southmead Hospital, Bristol	☐	☐
8	Cossham Birth Centre, Bristol	☐	☐
9	Mendip Birth Suite, Bristol	☐	☐
10	Scarborough Hospital, Scarborough	☐	☐
11	York hospital, York	☐	☐
12	Hull Royal Infirmary, Hull	☐	☐
13	Wonford Hospital, Exeter	☐	☐
14	Derriford Hospital, Plymouth	☐	☐
15	Kings Mill Hospital, Sutton-in-Ashfield	☐	☐
16	Liverpool Women's, Liverpool	☐	☐
17	Whiston Hospital, Merseyside	☐	☐
18	Countess of Chester Hospital, Chester	☐	☐
19	Westmorland General Hospital, Kendal	☐	☐
20	Royal Lancaster Infirmary, Lancaster	☐	☐
21	Furness General Hospital, Barrow-In-Furness	☐	☐
22	Doncaster Royal Infirmary, Doncaster	☐	☐
23	Bassetlaw Hospital, Worksop	☐	☐
24	Pinderfields Hospital, Wakefield	☐	☐
25	Dewsbury Hospital, Dewsbury	☐	☐
26	Pontefract Hospital, Pontefract	☐	☐
27	John Radcliffe Hospital, Headington	☐	☐
28	Horton General Hospital, Banbury	☐	☐
29	Oxford Spires Birth Centre, Headington	☐	☐
30	Birmingham Women’s Hospital, Birmingham	☐	☐
31	Queen Elizabeth Hospital, Gateshead	☐	☐

2 **In general, how do you feel about the care you received during your pregnancy?**

a) Very positive	b) Quite positive	c) Slightly positive	d) No feelings	e) Slightly negative	f) Quite negative	g) Very negative
☐	☐	☐	☐	☐	☐	☐

Was anything particularly good or bad?

……………………………………………………………………………………………………..

………………………………………………………………………………………………………

………………………………………………………………………………………………………

………………………………………………………………………………………………………

3 **In general, how do you feel about the care you received during labour?**

a) Very positive	b) Quite positive	c) Slightly positive	d) No feelings	e) Slightly negative	f) Quite negative	g) Very negative
☐	☐	☐	☐	☐	☐	☐

Was anything particularly good or bad?

………………………………………………………………………………………………………

………………………………………………………………………………………………………

………………………………………………………………………………………………………

………………………………………………………………………………………………………

4 **What mattered to you during your antenatal appointments?**

………………………………………………………………………………………………………

………………………………………………………………………………………………………

………………………………………………………………………………………………………

………………………………………………………………………………………………………

………………………………………………………………………………………………………

5 **Did you smoke at the beginning of this pregnancy?**

a) Yes ☐

b) No ☐ **(skip to question 8)**

c) Don’t remember ☐ **(skip to question 8)**

6 **How many cigarettes did you smoke in a typical day during your pregnancy?**

a) 1-10 ☐
b) 11-20 ☐
c) 21-30 ☐
d) 31-40 ☐
e) More than 40 ☐

7 **Were you referred to a stop smoking service?**

a) Yes, I attended ☐
b) Yes, but I did not attend ☐
c) No ☐
d) Don't remember ☐

If you did **not attend** a stop smoking service**,** please tell us about this:

……

……

……

8 **At your booking appointment (usually your first appointment) were you asked by your midwife whether you smoked cigarettes?**

a) Yes ☐
b) No ☐
c) I reported I did smoke/did not smoke before I was asked ☐
d) Don't remember ☐

9 **Were you offered a breath test to measure carbon monoxide at your antenatal booking appointment?**

a) Yes, I took the test ☐
b) Yes, but I declined the test ☐
c) Yes, but it was offered at another appointment ☐
d) No, I was not offered the test ☐ **(skip to question 11)**
e) Don't remember ☐ **(skip to question 11)**

9 **Continued:**

If you decided **not** to take the carbon monoxide breath test, please tell us about this:

………

………

………

………

10 **How did you feel about being offered a carbon monoxide breath test?**

………

………

………

………

11 **Did you smoke around the time you had your baby?**

a) Yes ☐

b) No ☐ **(skip to question 13)**

12 **How many cigarettes did you smoke in a typical day around the time you had your baby?**

a) 1-10 ☐

b) 11-20 ☐

c) 21-30 ☐

d) 31-40 ☐

e) More than 40 ☐

We would now like to know more about your baby's movements and how they were monitored:

13 **Were you given this Reduced Fetal Movements leaflet (produced by Tommy’s/NHS England) about your baby’s movements?**

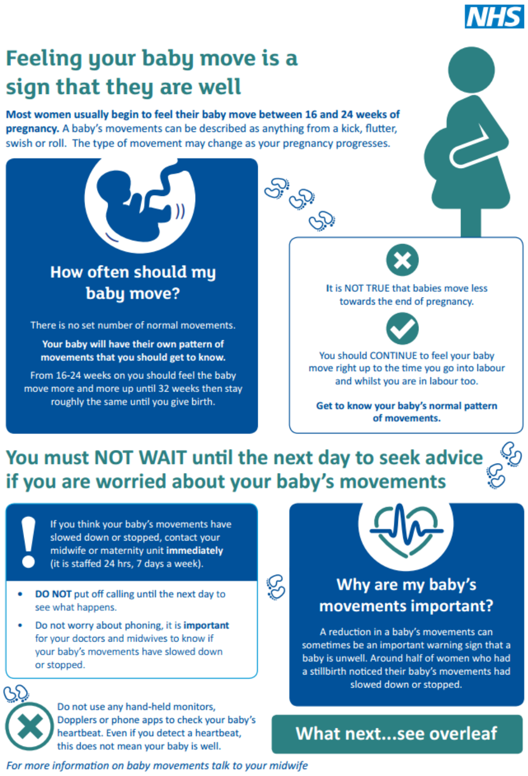
NHS

Feeling your baby move is a sign that they are well

Most women usually begin to feel their baby move between 16 and 24 weeks of pregnancy. A baby’s movements can be described as anything from a kick, flutter, swish or roll. The type of movement may change as your pregnancy progresses.

How often should my baby move?

There is no set number of normal movements.

Your baby will have their own pattern of movements that you should get to know.

From 16-24 weeks on you should feel the baby move more and more up until 32 weeks then stay roughly the same until you give birth.

It is NOT TRUE that babies move less towards the end of pregnancy.

You should CONTINUE to feel your baby move right up to the time you go into labour and whilst you are in labour too.

Get to know your baby’s normal pattern of movements.

You must NOT WAIT until the next day to seek advice if you are worried about your baby’s movements

If you think your baby’s movements have slowed down or stopped, contact your midwife or maternity unit **immediately** (it is staffed 24 hrs, 7 days a week).

- **DO NOT** put off calling until the next day to see what happens.
- Do not worry about phoning, it is **important** for your doctors and midwives to know if your baby’s movements have slowed down or stopped.

Why are my baby’s movements important?

A reduction in a baby’s movements can sometimes be an important warning sign that a baby is unwell. Around half of women who had a stillbirth noticed their baby’s movements had slowed down or stopped.

Do not use any hand-held monitors, Dopplers or phone apps to check your baby’s heartbeat. Even if you detect a heartbeat, this does not mean your baby is well.

What next...see overleaf

For more information on baby movements talk to your midwife

a) Yes, I read the leaflet ☐

b) Yes, but I did not read the leaflet ☐

c) No, I was given a different leaflet on baby’s movements ☐

d) No, I was not given a leaflet ☐ **(skip to question 15)**

e) Don’t remember ☐ **(skip to question 15)**

If you were given a different leaflet, please tell us the name:

……………………………………………………………………………………

……………………………………………………………………………………

……………………………………………………………………………………

……………………………………………………………………………………

14 **Was this given to you before you were 24 weeks pregnant?**

a) Yes, at my booking appointment ☐

b) Yes, at another appointment ☐

c) No ☐

d) Don’t remember ☐

15 **Did your midwife discuss your baby’s movements with you and what to do if they slowed/stopped during your pregnancy?**

a) Yes, at every antenatal appointment ☐

b) Yes, but only at one or some antenatal appointments ☐

c) No ☐

d) Don’t remember ☐

16 **Did you monitor your baby’s movements during this pregnancy?**

a) Yes, throughout my pregnancy ☐

b) Yes, through most of my pregnancy ☐

c) Yes, through some of my pregnancy ☐

16 **Continued:**

d) No ☐ **Skip to question 22**

Please tell us about this:

………

………

………

………

17 **What made you decide to monitor your baby's movements during this pregnancy? (tick all that apply)**

a) The "Reduced Fetal Movements" leaflet (produced by Tommy's/NHS England) ☐

b) A different leaflet on reduced fetal movements ☐

c) Advice from a maternity healthcare professional (e.g. midwife, doctor) ☐

d) Advice from the internet ☐

e) Advice from family or friends ☐

f) Nothing ☐

g) Other, **please specify**: ☐

……………………………………………………………………………………………

……………………………………………………………………………………………

……………………………………………………………………………………………

18 **How did you monitor your baby's movements? (tick all that apply)**

a) Stayed vigilant of movements ☐

b) Used an online App ☐

c) Used a Kick Chart ☐

d) Other, **please specify** ☐

………

………

………

………

………

19 **Did anything help you monitor your baby's movements?**

....

....

....

....

20 **Did anything make it difficult for you to monitor your baby's movements?**

....

....

....

....

21 **How did monitoring your baby's movements make you feel during pregnancy:**

a) Very calm	b) Slightly calm	c) Had no effect	d) Slightly anxious	e) Very anxious
☐	☐	☐	☐	☐

22 **Were you concerned at any point during this pregnancy that your baby's movements had slowed/stopped?**

a) Yes ☐ How many weeks pregnant were you when this happened?

....

b) No ☐ **(skip to question 26)**

c) Don't remember ☐ **(skip to question 26)**

23 **Did you contact a midwife when your baby's movements slowed/stopped?**

a) Yes, immediately ☐

b) Yes, but not straightaway ☐

c) Yes, but unable to speak to a midwife ☐

d) No ☐ **If not, please tell us about this:**

....

24 **Did you visit your maternity unit when your baby's movements slowed/stopped?**

a) Yes ☐

b) No ☐ **If not, please tell us about this: (skip to question 26)**

……………………………………………………………………………………

……………………………………………………………………………………

……………………………………………………………………………………

……………………………………………………………………………………

25 **What happened when you visited the maternity unit? (tick all that apply)**

a) A healthcare professional listened to the baby's heartbeat ☐

b) A healthcare professional took a trace of the baby's heart ☐

c) A healthcare professional performed an ultrasound scan ☐

d) A healthcare professional recommended delivery ☐

e) No action was taken ☐

f) Other, **please specify** ☐

……………………………………………………………………………………

……………………………………………………………………………………

……………………………………………………………………………………

……………………………………………………………………………………

……………………………………………………………………………………..

26 **How do you feel about the amount of information given to you about your baby's movements during pregnancy?**

a) I was given too much information ☐

b) I was given the right amount of information ☐

c) I was not given enough information ☐

27 **Which ONE way would you prefer to be given information about baby's movements?**

a) Verbally by a healthcare professional ☐

b) A leaflet ☐

c) A website ☐

d) Other ☐ **Please specify:**

…………………………………………………………………

…………………………………………………………………

28 **Are you aware of the 'Kicks Count' campaign to increase awareness of monitoring babies' movements during pregnancy?**

a) Yes ☐ **How did you hear about it?**

………

………

b) No ☐

Please turn over the page

Demographics

Please provide some information about yourself.

29 Ethnic origin?

WHITE

a) British ☐

b) Irish ☐

m) Other ☐ **Please specify**

…………………………………………………………………

MIXED

c) White and Black Caribbean ☐

d) White and Black African ☐

e) White and Asian ☐

m) Other ☐ **Please specify**

…………………………………………………………………

ASIAN OR ASIAN BRITISH

f) Indian ☐

g) Pakistani ☐

h) Bangladeshi ☐

m) Other ☐ **Please specify**

…………………………………………………………………

BLACK OR BLACK BRITISH

i) Caribbean ☐

j) African ☐

m) Other ☐ **Please specify**

…………………………………………………………………

OTHER ETHNIC GROUPS

k) Chinese ☐

m) Other ☐ **Please specify**

…………………………………………………………

l) Prefer not to say ☐

30 Age (years)?

……………………………………………..

31 **Do you consider yourself to have a disability?**

a) Yes ☐ **Please specify**

....

b) No ☐

32 **Is your first language English?**

a) Yes ☐

b) No ☐ **My first language is**

....

33 **What is your marital status?**

a) Married ☐

b) Living together ☐

c) Living separately ☐

d) Single ☐

e) Divorced / Separated ☐

f) Widowed ☐

g) Prefer not to say ☐

34 **What is your highest level of education?**

a) None ☐

b) Primary school ☐

c) GCSE / O-levels / Scottish Standard Grades / NVQ / BTEC ☐

d) A-levels / Scottish Highers and Advanced Highers / IB ☐

e) Undergraduate degree (e.g. Bachelor's) ☐

f) Postgraduate degree (e.g. Master's/PGCE) ☐

g) Doctorate ☐

35 **Employment status: Are you typically…?**
If you are currently on maternity leave, please answer this question with your employment status before you went on maternity leave.

a) Employed for wages ☐

b) Self-employed ☐

c) Out of work and looking for work ☐
d) Out of work and <u>not</u> looking for work ☐
e) A student ☐
f) A homemaker ☐
g) Retired ☐
h) Unable to work ☐

36 **How old is your baby (days)?**

…………………………………………

37 **How did you give birth to your baby?**

a) Normal vaginal delivery ☐
b) Ventouse delivery (kiwi cup/ suction cup) ☐
c) Forceps delivery ☐
d) Emergency caesarean section ☐
e) Elective (planned) caesarean section ☐

38 **Did you attend the hospital for any extra appointments during your pregnancy?**

a) Yes ☐ **Please specify:**

……………………………………………………………………………………..

……………………………………………………………………………………..

……………………………………………………………………………………..

b) No ☐

39 **Did you experience any of the following during this pregnancy? (tick all that apply)**

a) Reduced fetal movements ☐
b) Vaginal bleeding ☐
c) Baby was small ☐
d) Premature delivery ☐
e) None ☐

40 **How many children do you have (including this baby)?**

…………………………………………………………………….

41 **If you have been pregnant before this pregnancy, did you experience any of the followingcomplications in your previous pregnancy/ies? (tick all that apply)**

a) Baby was small ☐

b) Miscarriage ☐

c) Stillbirth ☐

d) Early neonatal death (baby died before leaving the hospital) ☐

e) N/A ☐

42 **Who did you see for your antenatal care and during labour and delivery?**

a) I only saw doctors ☐

b) I saw doctors and midwives ☐

c) I only saw midwives ☐

THANK YOU VERY MUCH FOR YOUR HELP

Please check that you have answered all of the questions that apply to you.

Please return the completed questionnaire to your midwife or a staff member.

MANCHESTER
1824
The University of Manchester

Saving Babies Lives Project Impact and Results Evaluation (SPIRE)

This study is being conducted by the University of Manchester on behalf of NHS England.

IRAS 223553, REC 17/WM/0197

Patient questionnaire, Version 2.4 12 June 2017

Contact Details

This section is entirely optional.

Your contact details will be kept separately from your answers and therefore, the researchers will not be able to identify you from the answers you provide.

If you would like to be entered into the prize draw to win a **£100 Amazon voucher**, please provide your name and contact details below and tick this box:

If you would like to receive a summary of the research findings once the study has ended, please provide your name and contact details below and tick this box:

You should only provide the information if you are happy to be contacted in that way. For example, if you do not want to be contacted by phone, do not provide a phone number.

You do not have to give your contact details if you do not want to be contacted for either reason.

Name

………………………………………………………………………………………

Email address

………………………………………………………………………………………

Telephone number*

………………………………………………………………………………………

* We will telephone you if you win the prize and have provided your telephone number. We will send you a text message if you would like to receive a summary of the findings and have provided your telephone number.
