# Supplementary material for: Stillbirth rates, service outcomes and costs of implementing NHS England’s Saving Babies’ Lives care bundle in maternity units in England: A cohort study
Source: PLoS One. 2021 Apr 19;16(4):e0250150. doi: 10.1371/journal.pone.0250150 (PMC8055032; doi:10.1371/journal.pone.0250150)
Supplement: S2 File — (PDF) [file pone.0250150.s006.pdf]

Staff Survey - Maternity Services

What is this survey about?

You are being invited to take part in a questionnaire being run by the University of Manchester on behalf of NHS England. You have been invited because you are currently a healthcare professional and have been working in your NHS hospital prior to April 2015. The questionnaire will help us better understand the impact that the Saving Babies' Lives Care Bundle has had on your own practice and the services provided by your maternity unit.

What is my information used for?

Your responses will help us evaluate how antenatal care is delivered following the implementation of the Saving Babies' Lives Care Bundle and will then be used by the NHS to improve antenatal care across the country.

What do I need to do?

- Firstly, the questionnaire will ask you about how antenatal care is currently delivered at your hospital in relation to the Saving Babies' Lives Care Bundle. Some of the questions will be multiple-choice and others will ask you to give some more details about your thoughts and experiences.
- Secondly, it will ask you to answer some demographic questions, such as your age and role.
- The survey should take approximately 15-30 minutes to complete.

Can I withdraw my answers from the study?

Because the questionnaire is completed anonymously (the researchers cannot identify you), once you submit your answers, you will **not** be able to withdraw them. Therefore, by completing this questionnaire you understand that your answers cannot be withdrawn and they will be used in this study. Please do not complete the questionnaire if you are not happy with this.

Will I receive anything for completing this survey?

Once you have completed the questionnaire, you will be given the option to provide your contact details for two reasons:

1) To enter you into a prize draw to win a **£100 Amazon voucher** as a thank you for completing the questionnaire. If you provide your contact details for this reason we will only use your details to contact you if you have won the prize.
2) If you wish to receive a summary of the research findings once the study has ended.

Your contact details will be kept separately from your answers and therefore, the researchers will not be able to identify you from the answers you provide. You do not have to provide your contact details if you do not want to.

What will happen to the information I supply?

The information you supply will be analysed by the University of Manchester's research team. Once the study has ended we will keep your anonymous answers for 5 years in line with University of Manchester's recommendations. The information you supply may be viewed by responsible individuals from the University of Manchester and regulatory authorities. Any contact details you provide will only be kept until the end of the study and then they will be destroyed. We will not pass them on to any third parties.

Can I get more information about the study?

If you would like more information about your participation in the study, have any questions, or would like a summary of the findings once the study has ended (instead of providing your contact details), please don't hesitate to contact:

Dr Kate Widdows (Project Manager)
Maternal and Fetal Health Research
5th Floor Research, St. Mary's Hospital
Oxford Road
Manchester
M13 9WL
Telephone: (0161) 276 6916
kate.widdows@manchester.ac.uk

Dr Alexander Heazell (Senior Clinical Lecturer)
Maternal and Fetal Health Research
5th Floor Research, St. Mary's Hospital
Oxford Road
Manchester
M13 9WL
Telephone: (0161) 701 0889
alexander.heazell@manchester.ac.uk

You do not have to complete the questionnaire if you do not want to.

Please answer the following questions about your role:

1 In which unit (or for which Trust, if community midwife) do you currently work?

1 Musgrove Park Hospital, Taunton ☐
2 Barnsley Hospital, Barnsley ☐
3 Cumberland Infirmary, Carlisle ☐
4 West Cumberland Hospital, Whitehaven ☐
5 St. Mary's Hospital, Manchester ☐
6 Royal United Hospital, Bath ☐
7 Southmead Hospital, Bristol ☐
8 Cossham Birth Centre, Bristol ☐
9 Mendip Birth Suite, Bristol ☐
10 Scarborough Hospital, Scarborough ☐
11 York Hospital, York ☐
12 Hull Royal Infirmary, Hull ☐
13 Wonford Hospital, Exeter ☐
14 Derriford Hospital, Plymouth ☐
15 Kings Mill Hospital, Sutton-in-Ashfield ☐
16 Liverpool Women's, Liverpool ☐
17 Whiston Hospital, Merseyside ☐
18 Countess of Chester Hospital, Chester ☐
19 Westmorland General Hospital, Kendal ☐
20 Royal Lancaster Infirmary, Lancaster ☐
21 Furness General Hospital, Barrow-In-Furness ☐
22 Doncaster Royal Infirmary, Doncaster ☐
23 Bassetlaw Hospital, Worksop ☐
24 Pinderfields Hospital, Wakefield ☐
25 Dewsbury Hospital, Dewsbury ☐
26 Pontefract Hospital, Pontefract ☐
27 John Radcliffe Hospital, Headington ☐
28 Horton General Hospital, Banbury ☐
29 Oxford Spires Birth Centre, Headington ☐
30 Birmingham Women’s Hospital, Birmingham ☐
31 Queen Elizabeth Hospital, Gateshead ☐

Community Midwives only:

32 Royal Devon & Exeter NHS Foundation Trust ☐
33 Plymouth Hospital NHS Trust ☐
34 Sherwood Forest Hospitals NHS Foundation Trust ☐
35 Liverpool Women's NHS Foundation Trust ☐
36 St H&K Teaching Hospitals NHS Trust ☐
37 Countess of Chester Hospital NHS Foundation Trust ☐
38 University Hospitals of Morecambe Bay NHS Foundation Trust ☐
39 Doncaster and Bassetlaw Hospitals NHS Foundation Trust ☐
40 The Mid Yorkshire Hospitals NHS Trust ☐
41 Oxford University Hospitals NHS Trust ☐
42 Birmingham Women's NHS Foundation Trust ☐
43 Gateshead Health NHS Foundation Trust ☐
44 Taunton and Somerset NHS Foundation Trust ☐
45 Barnsley Hospital NHS Foundation Trust ☐
46 North Cumbria University Hospitals NHS Trust ☐
47 Central Manchester University Hospitals Foundation Trust ☐
48 Royal United Hospitals Bath NHS Foundation Trust ☐
49 North Bristol NHS Trust ☐
50 York Teaching Hospital NHS Foundation Trust ☐
51 Hull and East Yorkshire Hospital NHS Trust ☐

2 **What is your primary work area(s) (clinical area or department in which you spend most of your work time/provide most of your clinical services) in the maternity unit? (tick all that apply)**

a) Antenatal clinic ☐
b) Antenatal ward ☐
c) Labour ward/Birthing suite ☐
d) Postnatal ward ☐
e) Community ☐
f) Ultrasound department ☐
g) Fetal medicine department ☐
h) Practice education ☐
i) Other, **please specify:** ☐

………

3 **How long have you been working in this particular work area (years and months)?**

....

4 **Do you regularly provide antenatal care?**

a) Yes ☐

b) No ☐ **(skip to question 13)**

5 **Do you offer women a carbon monoxide (CO) breath test during antenatal appointments?**

a) Yes ☐

b) No ☐ **(skip to question 8)**

c) Don't remember ☐ **(skip to question 8)**

6 **How do you offer the CO breath test to women?**

a) As a general test of a woman's wellbeing ☐

b) Specifically as an assessment of a woman's smoking status ☐

c) Other, **please specify:** ☐

....

7 **Does your work area have enough CO monitors to ensure all women are offered the breath test?**

a) Yes ☐

b) No ☐

c) Don't know ☐

8 **How do you assess a woman's smoking status? (tick all that apply)**

a) Verbally ☐

b) The woman completes a self-report form ☐

c) A CO breath test ☐

d) A woman's smoking status is **not** assessed ☐ **(skip to question 10)**

e) Other, **please specify:** ☐

....

9 **Do you record a woman's smoking status at antenatal booking?**

a) Yes ☐

b) No ☐ **If not, please tell us why:**

……………………………………………………………………………………………

……………………………………………………………………………………………

10 **How did you feel about administering a CO breath test to women?**

……………………………………………………………………………………………

……………………………………………………………………………………………

……………………………………………………………………………………………

11 **Do you refer women to stop smoking service if they do smoke or have a positive CO reading?**

a) Yes ☐ **Which service(s)?**

……………………………………………………………………………………………

……………………………………………………………………………………………

b) No ☐ **If not, please tell us why:**

……………………………………………………………………………………………

……………………………………………………………………………………………

12 **Do you give women the Reduced Fetal Movements leaflet (produced by Tommy's/NHS England) before 24 weeks gestation? (tick all that apply)**

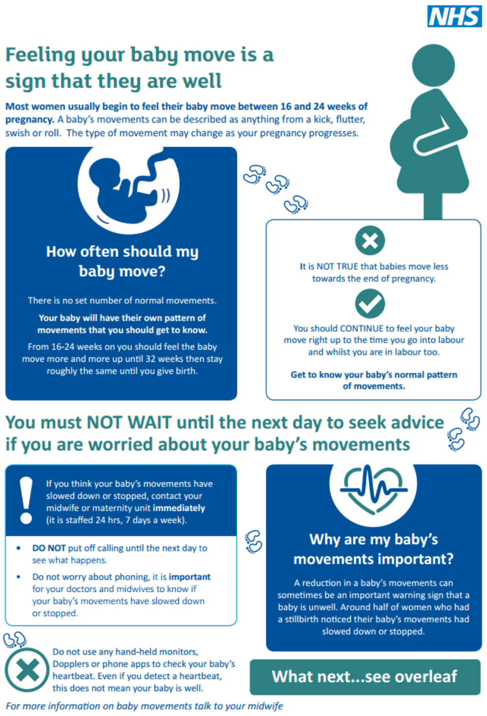

a) Yes ☐

b) No, I give women a different leaflet on reduced fetal movements. ☐
Please specify:

……………………………………………………………………………

……………………………………………………………………………

c) No, I do not give women any leaflet on reduced fetal movements ☐
Please tell us why:

……………………………………………………………………………

……………………………………………………………………………

d) Don't know about this leaflet ☐

13 **Do you deliver care to women when they report reduced fetal movements?**

a) Yes ☐

b) No ☐ **(skip to question 15)**

14 **Do you use the Saving Babies' Lives *Checklist for Management of Reduced Fetal Movements* to manage the care of women who report reduced fetal movements over 28 weeks of pregnancy?**

a) Yes ☐

b) No, I use another checklist ☐ **Please specify:**

……………………………………………………………………………………

……………………………………………………………………………………

c) No, I don't use a checklist ☐ **Please tell us why:**

……………………………………………………………………………………

……………………………………………………………………………………

15 **Are you involved in assessing fetal growth (through ultrasound scanning and/or symphysis fundal height measurements)?**

a) Yes ☐

b) No ☐ **(skip to question 23)**

16 **Do you use the Saving Babies' Lives algorithm to classify a woman's risk of fetal growth restriction?**

a) Yes ☐

b) No, I use a different algorithm ☐ **Which algorithm do you use?**

……………………………………………………………………………

c) No, I don't use any algorithm ☐

d) Don't know ☐

17 **If a woman's pregnancy is classified as high risk for fetal growth restriction, what gestation are ultrasound scans usually performed? (e.g. 28 weeks, 36 weeks)**

………

………

………

………

18 **How do you perceive the demand on the ultrasound scanning at your hospital?**

a) Too high ☐
b) Very high ☐
c) High ☐
d) Manageable ☐
e) Low ☐
f) Very low ☐
g) Too low ☐
h) Don't know ☐

19 **Do you assess fetal growth using symphysis fundal height customised charts?**

a) Yes ☐
b) No ☐ **(skip to question 23)**

20 **Do you feel adequately trained in measuring symphysis fundal height?**

a) Yes, no need for more training ☐
b) Yes, but would like more training ☐
c) No ☐

Comments on this training (optional):

………

………

………

21 **Do you feel adequately trained in plotting symphysis fundal height measurements on customised charts?**

a) Yes, no need for more training ☐
b) Yes, but would like more training ☐
c) No ☐

Comments on this training (optional):

………

………

………

22 **Who provided your training in the use of symphysis fundal height charts? (tick all that apply)**

a) A GROW (Gestation Related Optimal Weight) trainer who used GROW materials e.g. The Perinatal Institute ☐

b) Other **(Please specify)** ☐

....

c) Not training received ☐

23 **Do you care for women in labour?**

a) Yes ☐

b) No ☐ **(skip to question 32)**

24 **Have you taken an annual training and competency assessment on cardiotocograph (CTG) interpretation in the last 12 months? (tick all that apply)**

a) Yes, annual training ☐ **Who provided the training?**

....

....

b) Yes, competency assessment ☐

c) Neither ☐

d) Don't know ☐

Comments on this training (optional):

....

....

....

....

25 **Do you feel competent in CTG interpretation?**

a) Yes, completely competent ☐

b) Yes, but I would like more training ☐

c) No ☐

26 **MIDWIVES ONLY – Have you taken an annual training and competency assessment on the use of intermittent auscultation? (tick all that apply)**

a) Yes, annual training ☐ **Who provided the training?**

……………………………………………………………………………...

……………………………………………………………………………...

b) Yes, competency assessment ☐

c) Neither ☐

d) Don't know ☐

Comments on this training (optional):

……

……

……

……

27 **MIDWIVES ONLY - Do you feel competent in the use of intermittent auscultation?**

a) Yes, completely competent ☐

b) Yes, but I would like more training ☐

c) No ☐

28 **Does your hospital currently operate a 'buddying system' for the review of CTG interpretation?**

('Buddying system' = Midwives are paired with a 'buddy' midwife on each shift, and each of the pair must countersign in agreement with each other's categorisation of their CTG traces. If disagreement occurs, an escalation protocol is followed).

a) Yes ☐

b) No ☐

c) Don't know ☐

29 **How do you feel about a buddying system for the review of CTG interpretation?**

……

……

……

……

……

……

30 **How do you assess smoking status at delivery? (tick all that apply)**

a) Verbally ☐
b) The woman completes a self-report form ☐
c) A CO breath test ☐
d) A woman's smoking status is not assessed ☐ **(skip to question 32)**
e) Other (Please specify) ☐

……………………………………………………………………………………………………….....

31 **How is smoking status at delivery documented? (tick all that apply)**

a) In the patient's hand-held notes ☐
b) Electronically in the maternity information system ☐
c) Smoking status at delivery is not recorded ☐
d) Don't know ☐
e) Other (Please specify) ☐

……………………………………………………………………………………………………….....

32 **Over the last 5 years or since you have been working in your current work area, do you perceive that:**

i) The demand on the ultrasound scanning has

a) Greatly increased	b) Slightly increased	c) Not changed	d) Slightly decreased	e) Greatly decreased	f) I don't know
☐	☐	☐	☐	☐	☐

ii) The number of stillbirths has

a) Greatly increased	b) Slightly increased	c) Not changed	d) Slightly decreased	e) Greatly decreased	f) I don't know
☐	☐	☐	☐	☐	☐

iii) The number of babies admitted to a neonatal intensive care unit has

a) Greatly increased	b) Slightly increased	c) Not changed	d) Slightly decreased	e) Greatly decreased	f) I don't know
☐	☐	☐	☐	☐	☐

iv) The number of inductions has

a) Greatly increased	b) Slightly increased	c) Not changed	d) Slightly decreased	e) Greatly decreased	f) I don't know
☐	☐	☐	☐	☐	☐

v) The number of caesareans-sections has

a) Greatly increased	b) Slightly increased	c) Not changed	d) Slightly decreased	e) Greatly decreased	f) I don't know
☐	☐	☐	☐	☐	☐

33 **In my work area:**

i) Outcomes for the mother and baby are

a) Greatly improving	b) Slightly improving	c) Not changing	d) Slightly worsening	e) Greatly worsening	f) I don't know
☐	☐	☐	☐	☐	☐

ii) We have enough staff to ensure the best possible outcomes for the mother and baby

a) Completely agree	b) Agree	c) Neutral	d) Disagree	e) Completely disagree	f) I don't know
☐	☐	☐	☐	☐	☐

iii) We have enough equipment to ensure the best possible outcomes for the mother and baby

a) Completely agree	b) Agree	c) Neutral	d) Disagree	e) Completely disagree	f) I don't know
☐	☐	☐	☐	☐	☐

iv) We are actively doing things to improve the safety of mothers and babies

a) Completely agree	b) Agree	c) Neutral	d) Disagree	e) Completely disagree	f) I don't know
☐	☐	☐	☐	☐	☐

v) The safety of the mother and baby are sacrificed to get more work done

a) Completely agree ☐ b) Agree ☐ c) Neutral ☐ d) Disagree ☐ e) Completely disagree ☐ f) I don't know ☐

vi) Management seem interested in safety only after an adverse event happens

a) Completely agree ☐ b) Agree ☐ c) Neutral ☐ d) Disagree ☐ e) Completely disagree ☐ f) I don't know ☐

vii) Staff work longer hours than is best for the mother and baby

a) Completely agree ☐ b) Agree ☐ c) Neutral ☐ d) Disagree ☐ e) Completely disagree ☐ f) I don't know ☐

We would like to know about your opinions of clinical guidelines in general and their use in clincial practice. Please answer questions 34, 35, 36 and 37 in relation to clinical guidelines in general, not specifically in relation to the Saving Babies' Lives Care Bundle guidelines.

34 By following clinical practice guidelines in my clinical decision-making:

i) I offer women higher-quality care

a) Completely agree ☐ b) Agree ☐ c) Neutral ☐ d) Disagree ☐ e) Completely disagree ☐ f) I don't know ☐

ii) I ensure that all women receive the same level of basic care

a) Completely agree ☐ b) Agree ☐ c) Neutral ☐ d) Disagree ☐ e) Completely disagree ☐ f) I don't know ☐

35 **How do the following individuals view the use of clinical guidelines?**

i) Your manager

a) Definitely should use ☐
b) Probably should use ☐
c) Neutral ☐
d) Probably should not use ☐
e) Definitely should not use ☐
f) I don't know ☐

ii) Your work colleagues

a) Definitely should use ☐
b) Probably should use ☐
c) Neutral ☐
d) Probably should not use ☐
e) Definitely should not use ☐
f) I don't know ☐

36 **To what extent do you agree/disagree with the following statements regarding the use of clinical guidelines in antenatal and intrapartum care?**

i) A lack of time greatly impedes the use of guidelines in my unit

a) Completely agree ☐
b) Agree ☐
c) Neutral ☐
d) Disagree ☐
e) Completely disagree ☐
f) I don't know ☐

ii) Poor readability of guidelines of greatly impedes the use of guidelines in my unit

a) Completely agree ☐
b) Agree ☐
c) Neutral ☐
d) Disagree ☐
e) Completely disagree ☐
f) I don't know ☐

iii) I am not able to carry out all recommendations in the guidelines

a) Completely agree ☐
b) Agree ☐
c) Neutral ☐
d) Disagree ☐
e) Completely disagree ☐
f) I don't know ☐

iv) It is easy for me to access the relevant guideline when I need it

a) Completely agree ☐
b) Agree ☐
c) Neutral ☐
d) Disagree ☐
e) Completely disagree ☐
f) I don't know ☐

37 **How regularly do you refer to clinical guidelines to inform your practice?**

a) Daily ☐
b) Weekly ☐
c) Monthly ☐
d) Yearly ☐
e) Other ☐ **Please specify:**

…………………………………………………………………………………

38 **Are you aware of the Saving Babies' Lives Care Bundle?**

a) Yes ☐
b) No ☐ **(skip to question 44)**

39 **How were you made aware of the Saving Babies' Lives Care Bundle?**

………

………

………

………

40 **What has been the main impact of the Saving Babies' Lives Care Bundle on <u>your own practice</u>?**

………

………

………

41 **What has been the main impact of the Saving Babies' Lives Care Bundle on <u>your unit</u>?**

………

………

………

………

42 **What do you think is good/effective about the Saving Babies' Lives Care Bundle?**

....

....

....

....

....

43 **What would you change about the Saving Babies Lives' Care Bundle?**

....

....

....

....

....

44 **In your personal opinion, what do you feel could be changed to reduce stillbirths and early neonatal death?**

....

....

....

....

....

Please turn over the page

Demographics

Please provide some information about yourself

45 **Ethnic origin?**

WHITE

a) British ☐

b) Irish ☐

m) Other ☐ **Please specify**

……………………………………………………………………

MIXED

c) White and Black Caribbean ☐

d) White and Black African ☐

e) White and Asian ☐

m) Other ☐ **Please specify**

……………………………………………………………………

ASIAN OR ASIAN BRITISH

f) Indian ☐

g) Pakistani ☐

h) Bangladeshi ☐

m) Other ☐ **Please specify**

……………………………………………………………………

BLACK OR BLACK BRITISH

i) Caribbean ☐

j) African ☐

m) Other ☐ **Please specify**

…………………………………………………………………..

OTHER ETHNIC GROUPS

k) Chinese ☐

m) Other ☐ **Please specify**

…………………………………………………………………..

l) Prefer not to say ☐

46 **Age (years)?**

……

47 **Gender?**

a) Male ☐

b) Female ☐

c) Prefer not to say ☐

48 **What is your current post?**

a) Consultant Obstetrician ☐

b) Consultant in Fetomaternal Medicine ☐

c) Speciality Trainee 6-7 ☐

d) Speciality Trainee 3-5 ☐

e) Speciality Trainee 1-2 ☐

f) Subspecialty Trainee in Fetomaternal Medicine ☐

g) Head of Midwifery ☐

h) Midwife Band 8 (**not** regularly working in the community) ☐

i) Midwife Band 8 (regularly working in the community) ☐

j) Midwife Band 7 (**not** regularly working in the community) ☐

k) Midwife Band 7 (regularly working in the community) ☐

l) Midwife Band 6 (**not** regularly working in the community) ☐

m) Midwife Band 6 (regularly working in the community) ☐

n) Midwife Band 5 (**not** regularly working in the community) ☐

o) Midwife Band 5 (regularly working in the community) ☐

p) Ultrasonographer Band 8 ☐

q) Ultrasonographer Band 7 ☐

r) Ultrasonographer Band 6 ☐

s) Other (Please specify)

....

49 **How long have you been in your current post (years and months)?**

....

50 **How long have you been qualified to deliver antenatal care and/or care during labour (years and months)?**

…………………………………………………………………………………………

51 **For how long have you delivered maternity care in the unit (or in the Trust, if community midwife) in which you currently work (years/months)?**

…………………………………………………………………………………………

52 **Typically, how many hours per week do you work in your current role?**

…………………………………………………………………………………………

THANK YOU VERY MUCH FOR YOUR HELP

Please check that you have answered all of the questions that apply to you.

Please return the completed questionnaire to your unit's Saving Babies' Lives Care Bundle Evaluation representative.

MANCHESTER 1824
The University of Manchester

Saving Babies Lives Project Impact and Results Evaluation (SPIRE)

This study is being conducted by the University of Manchester on behalf of NHS England.

IRAS 223553, REC 17/WM/0197

Professionals questionnaire, Version 2.4, 27 June 2017

Contact Details

This section is entirely optional.

Your contact details will be kept separately from your answers and therefore, the researchers will not be able to identify you from the answers you provide.

If you would like to be entered into the prize draw to win a **£100 Amazon voucher**, please provide your name and contact details below and tick this box: ☐

If you would like to receive a summary of the research findings once the study has ended, please provide your name and contact details below and tick this box: ☐

You should only provide the information if you are happy to be contacted in that way. For example, if you do not want to be contacted by phone, do not provide a phone number**.**

You do not have to give your contact details if you do not want to be contacted for either reason.

Name

………………………………………………………………………………………

Email address

………………………………………………………………………………………

Telephone number*

………………………………………………………………………………………

* We will telephone you if you win the prize and have provided your telephone number. We will send you a text message if you would like to receive a summary of the findings and have provided your telephone number.
