# Supplementary material for: Stillbirth rates, service outcomes and costs of implementing NHS England’s Saving Babies’ Lives care bundle in maternity units in England: A cohort study
Source: PLoS One. 2021 Apr 19;16(4):e0250150. doi: 10.1371/journal.pone.0250150 (PMC8055032; doi:10.1371/journal.pone.0250150)
Supplement: S3 File — (PDF) [file pone.0250150.s007.pdf]

Supplementary File - Characteristics of participants in the questionnaire for health care professionals working within maternity units participating in the Saving Babies Lives Care Bundle (n=1,064).

**Characteristic**	**Response**	**Number (%)**
Maternity Unit		
	A	24 (2.3)
	B	17 (1.6)
	C	34 (3.2)
	D	58 (5.5)
	E	67 (6.3)
	F	50 (4.7)
	G	59 (5.5)
	H	70 (6.6)
	I	98 (9.2)
	J	43 (4.0)
	K	29 (2.7)
	L	31 (2.9)
	M	86 (8.1)
	N	51 (4.8)
	O	52 (4.9)
	P	42 (3.9)
	Q	126 (11.3)
	R	89 (8.4)
	S	38 (3.6)
Role within unit		
	Consultant	65 (6.1)
	Manager	3 (0.3)
	Midwife	830 (78.0)
	Nurse/Sister	9 (0.8)
	Other	10 (0.9)
	Trainee Doctor	15 (1.4)
	Ultrasonographer	34 (3.2)
	Not Stated	98 (9.2)
Regularly provide antenatal care		
	Yes	738 (69.4)
	No	316 (29.7)
	No Response	10 (0.9)
		**Median [IQR]**
Age	Years	45 [34-52]
Length of time working in current role	Years	7 [3-12]
Length of time since qualification	Years	13 [7-25]
