# Supplementary material for: Stillbirth rates, service outcomes and costs of implementing NHS England’s Saving Babies’ Lives care bundle in maternity units in England: A cohort study
Source: PLoS One. 2021 Apr 19;16(4):e0250150. doi: 10.1371/journal.pone.0250150 (PMC8055032; doi:10.1371/journal.pone.0250150)
Supplement: Attachment — Submitted filename: Response to Reviewer_SBLCB_Evaluation_Final.docx [file pone.0250150.s008.docx]

**Response to Reviewer’s Comments Manuscript: PONE-D-20-24240**

**Stillbirth rates, service outcomes and costs of implementing NHS England’s Saving Babies’ Lives care bundle in maternity units in England: a cohort study**

1. Please ensure that your manuscript meets PLOS ONE's style requirements, including those for file naming. The PLOS ONE style templates can be found at

<https://journals.plos.org/plosone/s/file?id=wjVg/PLOSOne_formatting_sample_main_body.pdf> and

<https://journals.plos.org/plosone/s/file?id=ba62/PLOSOne_formatting_sample_title_authors_affiliations.pdf>

 The manuscript has been formatted to match PLOS ONE’s style requirements.

2. In ethics statement in the manuscript and in the online submission form, please provide additional information about the patient records/samples used in your retrospective study. Specifically, please ensure that you have discussed whether all data/samples were fully anonymized before you accessed them and/or whether the IRB or ethics committee waived the requirement for informed consent. If patients provided informed written consent to have data/samples from their medical records used in research, please include this information.

All data were fully anonymized before analysis. Participants in the online survey were asked to indicate their consent to participation in their response.

3. In your Methods section, please provide additional information about the participant recruitment method and the demographic details of your participants. Please ensure you have provided sufficient details to replicate the analyses such as: a) the recruitment date range (month and year), b) a description of any inclusion/exclusion criteria that were applied to participant recruitment, c) a table of relevant demographic details, d) a statement as to whether your sample can be considered representative of a larger population, e) a description of how participants were recruited, and f) descriptions of where participants were recruited and where the research took place.

The Methods section includes the date ranges for specific components of the study. Table 1 describes the characteristics of the maternity units (NHS Trusts) that participated in the study. No demographic data for individual participants were collected. We have described how participating units were selected and where the research took place.

4. Please include additional information regarding the survey or questionnaire used in the study and ensure that you have provided sufficient details that others could replicate the analyses. For instance, if you developed a questionnaire as part of this study and it is not under a copyright more restrictive than CC-BY, please include a copy, in both the original language and English, as Supporting Information.

The questionnaire for women who gave birth at participating Trusts and the questionnaire for staff working at participating trusts have been included as supplementary files.

5. We noted in your submission details that a portion of your manuscript may have been presented or published elsewhere.

"This manuscript is original, but a portion of these data were presented previously in an archived report of the SPiRE evaluation which is available here –
<https://www.manchester.ac.uk/discover/news/download/573936/evaluationoftheimplementationofthesavingbabieslivescarebundleinearlyadopternhstrustsinenglandjuly2018-2.pdf>). "

Please clarify whether this publication was peer-reviewed and formally published. If this work was previously peer-reviewed and published, in the cover letter please provide the reason that this work does not constitute dual publication and should be included in the current manuscript.

This work was published on the University of Manchester website and a limited number of printed copies but was not peer-reviewed.

6. We note that you have indicated that data from this study are available upon request. PLOS only allows data to be available upon request if there are legal or ethical restrictions on sharing data publicly. For information on unacceptable data access restrictions, please see <http://journals.plos.org/plosone/s/data-availability#loc-unacceptable-data-access-restrictions>.

In your revised cover letter, please address the following prompts:

a) If there are ethical or legal restrictions on sharing a de-identified data set, please explain them in detail (e.g., data contain potentially identifying or sensitive patient information) and who has imposed them (e.g., an ethics committee). Please also provide contact information for a data access committee, ethics committee, or other institutional body to which data requests may be sent.

We did not ask participating units whether we could share the data they provided to the study. Therefore, we are not able to share it legally or under the terms of the research ethics approval granted for this study. Data requests should be sent to the Chief Investigator who can then approach the research ethics committee to determine whether access can be facilitated.

b) If there are no restrictions, please upload the minimal anonymized data set necessary to replicate your study findings as either Supporting Information files or to a stable, public repository and provide us with the relevant URLs, DOIs, or accession numbers. Please see <http://www.bmj.com/content/340/bmj.c181.long> for guidelines on how to de-identify and prepare clinical data for publication. For a list of acceptable repositories, please see <http://journals.plos.org/plosone/s/data-availability#loc-recommended-repositories>.

We will update your Data Availability statement on your behalf to reflect the information you provide.

7. Your ethics statement should only appear in the Methods section of your manuscript. If your ethics statement is written in any section besides the Methods, please delete it from any other section.

8. Please include captions for your Supporting Information files at the end of your manuscript, and update any in-text citations to match accordingly. Please see our Supporting Information guidelines for more information: [http://journals.plos.org/plosone/s/supporting-information](about:blank).

**Reviewer #1:**

General comments

In this manuscript the authors describe a cohort study used to perform and in-depth evaluation of a national initiative to address stillbirth rates in the UK. Although necessarily pragmatic in design, this is a well-constructed and reported study. The reported finding of a reduction in stillbirth rates across participating sites that exceeds that reported nationally in the same timeframe will be of interest to a broad, international readership.

Specific comments

Abstract

• Line 42, p 3- ‘Implementation of the SBL care bundle increased over time in the majority of sites with associated improvements in process outcomes. This statement lacks clarity and needs re- wording.

This statement has been re-worded and now reads “Implementation of the SBL care bundle increased over time in the majority of sites. Implementation was associated with improvements in process outcomes.” (lines 42-44)

Methods
• Line 114, p6- where authors describe method for scoring degree of implementation. What do you mean by totalled to give an implementation score for each element? Did each element have multiple components that were each scored then added or was there only one degree of implementation question per element? Was there any weighting given to certain components? i.e. some more important than others? It would be helpful for the reader to have clearly stated somewhere what ‘implementing’ element 1, 2, 3, 4, refers to (even though this may have been previously published).

The section on the implementation scoring has been revised for clarity. Units were asked to report a score for the implementation for each component of each element (ranked 0-3). These were then added to give the total for each element which is presented in Supplementary Figure 1. As some elements had different numbers of components the overall implementation was computed as the mean of each element expressed as a percentage of the total score (now edited lines 122-125). Thus all components were equally weighted within each element, and each element equally weighted in the overall implementation score. The authors have edited the manuscript to clarify what element 1, 2, 3 and 4 refers to in the introduction section (Lines 71-75) and also added the word “element” to caption of sup figure 1 to make this more explicit.

• Line 162, p9- Was there a specific reason staff time to complete training was not included in costs, especially for externally-provided training? Why were cost associated with neonatal outcomes not included? E.g. admission to NICU.

The time to complete training was not included in the primary estimate of costs due to the assumption that in-house training would be part of continuing professional development for staff. However, this has now been included in the sensitivity analyses reported in Table 6. Data on neonatal admissions were not included in the evaluation because of significant inconsistencies between data supplied from different providers which also meant that the associated costs could not be included in the economic analysis. The authors recognise that this is a limitation of the study (lines 335-336).

Discussion

• Line 267, p14- Authors mention a decrease in SB rate for ‘whole of England’, how much of this decrease can be accounted for by the trusts included in this evaluation? It may be worthwhile comparing SB rate reduction for ‘early adopters’ and ‘all other’ sites as separate groups as well as total ‘whole of England’ reduction?

The stillbirth rate for the whole of England across the duration of this study (as derived from central MBRRACE data) was 4.22 per 1,000 births in 2013 to 3.74 per 1,000 births in 2017. In participating units the rates fell from 4.23 per 1,000 births in 2013 to 3.40 per 1,000 births in 2017, in comparison units not participating in this evaluation the rates were 4.21 per 1,000 births in 2013 and 3.8 per 1,000 births in 2017. We have included this information in the discussion section. However, there were a number of sites not included in this evaluation that were implementing aspects of the SBL care bundle and one site (site S) within the evaluation who had very limited implementation. Therefore, we do not think it is appropriate to view this as a comparison between early adopters and all other sites.

Limitations

• Could the authors provide comment on whether self-reported degree of implementation as described is a limitation of the study and how this may have contributed to challenges if any with interpretation of results.

The authors agree with the reviewer that self-reported degree of implementation is a limitation of the study and this has been added to the limitations section of the study (lines 323-325). However, the process measures do give us an objective measure of implementation.

General

• Are the authors able to offer any further insights as to why adoption of the bundle was variable across sites and what unit characteristics made a ‘high degree’ implementer? E.g. influence of leadership, resources, other?

Unfortunately we did not collect information about the leadership structure or resources of individual Trusts to be able to make a valid judgement about whether these factors affected the degree of implementation of the care bundle across sites. We collected some information from staff about their perceptions of safety in the maternity unit where they work. This has been added to the manuscript. We have added this as an area for future work in the discussion section of the manuscript (lines 379-381).

• It would be of value to understand maternity staff attitudes towards the ‘bundle approach’, satisfaction with the way it was implemented and how this might have influenced process outcomes. Were there additional results from the survey of staff that are not reported here that might describe this?

We collected data from staff (n=1,064) regarding their views about the care bundle. We have previously reported staff views about clinical practice guidelines in their units (Lau et al. BMJ Open Quality 2020;9:e000756) and have not repeated these findings here. We have included information about staff views about the care bundle including the demand for ultrasound scanning, the stillbirth rate, induction rate and number of Caesarean sections in the results section. We have also included staff views about safety in their maternity unit (lines 261-267).

• Consider replacing the word “patients” with “women” and “deliveries” with “births” throughout.

Patients have been replaced with women and deliveries have been replaced with births throughout the manuscript.

Tables and Figures

• Table 3. – In source column consider replacing ‘Patient’ with ‘women”

Patient has been replaced by women in the source column of Table 3.

• Supplementary Figure 1- Heading check grammar- ‘implantaion’? Could the authors explain for section B why the x-axis has a different scale for each element? This goes back to previous comment in methods, it is unclear how you are calculating the score for each element. Could you also please explain why site K has an overall score of 0% yet does have scores for each element?

The heading has been altered to read “implementation”. An explanation has been added to explain why the x-axis is different for each element in the methods section. This has been reiterated in the figure legend. In figure A) implementation at baseline, implementation date and post-implementation is shown. In Figure B, the breakdown for the different elements is given at the post-implementation time point is shown. This is stated in the legend. The x-axis for the charts in section A has been altered to read % Implementation score.

• Given element 4 is intrapartum monitoring it would be helpful to see SB rate broken down into antepartum vs intrapartum to further explain changes in SB rate due to implementation of element 4.

Unfortunately we did not collect information on the timing of stillbirth so are not able to present the intrapartum stillbirth rate in our manuscript. However, the statistics from MBRRACE demonstrate that the intrapartum stillbirth rate fell from 0.32 per 1,000 births in participating units in 2013 to 0.06 per 1,000 births in 2016 (Relative risk 0.18, 95% CI 0.08, 0.45). As these data were not obtained from our data we have not included this information in the results section but have added it to the discussion section of the manuscript (lines 303-305).

**Reviewer #2:**

Thanks for allowing me to review your manuscript. I have several comments and questions.

1. Can you provide more details about the process for selecting the 19 NHS trusts involved in this evaluation? How was it that a trust with an implementation score of 0 was selected?

All Trusts that were deemed “early adopters” of the SBLCB in 2015 were eligible to take part; these were sites that completed the 2015 NHS England Tracker Survey indicating that they were implementing the SBLCB. Initially, Trusts were selected to take part in the evaluation to compare outcomes in providers reporting full, partial or low implementation stages as reported in the Tracker Survey, But at the time of the study in 2016 most Trusts had increased their implementation so the original study design of full, partial, low was not possibleUnits were purposively sampled to provide a geographically distributed sample throughout England and a mixture of Trusts with different numbers of births and levels of neonatal care. Units from London were not included as they were participating in a stepped-wedge cluster trial of implementation of the Growth Assessment Protocol (Trials 2019 Mar 4;20(1):154. doi: 10.1186/s13063-019-3242-6). The Trust (S) had an implementation score of 0 at baseline which improved to 40% post-implementation, so although the implementation from this site was far from that anticipated, some aspects of the SBL care bundle had been introduced.

Information regarding the selection of sites has been included in the methods section (lines 88-92).

2. I found the economic analysis difficult to follow due to the number of assumptions made. In particular, it seems that all of the increased interventions are costed as 'additional costs' whereas it may also be that increases in certain interventions such as induction of labour and caesarean section actually save money in terms of reducing other adverse outcomes. I do wonder if the economic analysis is worthy of a separate paper, which would then allow a much more comprehensive review of the costs of this bundle implementation.

We have rephrased the reporting of the results of the economic analysis to make it easier to follow. Due to the pragmatic and post hoc nature of the evaluation it was necessary to make a number of assumptions in order to estimate the costs associated with the care bundle as it was not possible to collect additional data. This also means that a more comprehensive review of the costs is not possible and so we feel that this combined paper is appropriate. We agree that there may have been some downstream cost-savings associated with fewer adverse outcomes, for example if there were fewer NICU admissions, however data were not available to quantify these impacts and so it was not possible to include them in this analysis. This has been added as a limitation in the discussion (lines 335-336).

3. The data presented gives rates of 'total stillbirths' and 'term stillbirths' but it would also be of interest to see how rates changed at lower gestations. It might be expected that the interventions would have a major impact on late gestation stillbirth but it appears that there have been come changes at earlier gestations as well. Can you provide that information? I am sure it would be of interest to the readers.

We do not have sufficiently detailed data to calculate the stillbirth rate for individual gestations or groups of gestational ages, but we are able to divide total stillbirths into “term stillbirths” and “preterm stillbirths”. The rate of term stillbirth decreased from 1.9 per 1,000 births pre-intervention to 1.4 per 1,000 births post-intervention (Relative Risk 0.77, p=0.005) and the rate of pre-term stillbirth decreased from 2.3 per 1,000 births to 1.9 per 1,000 births (Relative Risk 0.82, p=0.014). We have added this information to the manuscript to emphasise that there were reductions in both term and preterm stillbirths (lines 213-215).
