# Supplementary material for: Stillbirth rates, service outcomes and costs of implementing NHS England’s Saving Babies’ Lives care bundle in maternity units in England: A cohort study
Source: PLoS One. 2021 Apr 19;16(4):e0250150. doi: 10.1371/journal.pone.0250150 (PMC8055032; doi:10.1371/journal.pone.0250150)
Supplement: Attachment — Submitted filename: Response_to_Reviewer_260321.docx [file pone.0250150.s009.docx]

We would like to thank the reviewers for their comments on our manuscript. We note that Reviewer#2 has no further comments on our manuscript.

Reviewer #3: This is a well-conducted study; however its reporting does not follow the appropriate reporting guidelines, and I am unable to make out a number of key features; for example:

The study report was written according to the STROBE checklist. This was already uploaded as a supporting document to the PLoSOne website. We have included this information in the manuscript to ensure that readers are aware that we followed reporting guidelines (line 102).

- it is unclear whether the investigators obtained individual/participant level data or whether the data was collected already aggregated at trust or hospital hospital-level. I understand that it could have been a mix of these for different variables e.g. individual-level outcomes and trust-level covariates, however, even this needs to be made clear for the various variables when they are described.

The reviewer is correct that we used a mix of different methods of data collection for individual components of the analysis. We described these in the published protocol all these details are available to readers. We state in the methods the three different data structures

- Implementation data at Trust level (i.e. already aggregated data) (line 122)
- Clinical outcomes and smoking as monthly counts per centre (line 107ff)
- Audit data at specified time points (lines 111-114)
- Post-partum patient questionnaire (line 115 - 116). We have now clarified that this individual questionnaire data was only obtained in the post-intervention period (line 114).

Table 3 gives the source of data for each outcome. We have now made it explicit that “women” in table 3 refers to the questionnaire.

- the number of units studied should be reported very clearly when indicating the size of this study. For example, the abstract describes this as a retrospective cohort study of 463,630 births in 19 trusts and mentions 1,658 audits, an surveys of 2085 users and 1,064 health professionals, but it is still not quite clear which of these units the outcomes reported pertain to; for example, did you obtain stillbirth events for each participant or did you obtain them aggregated at hospital/trust level? It is also not clear how the numbers of units break down over the various times the assessments relate to.

Full details of the sources of each data are provided in the published protocol. The information on the numbers of units and women providing data for each outcome is provided in tables 2 and 3. The survey of women and questionnaires from health professionals were obtained from all 19 participating units. Data regarding the characteristics of staff participants has been added as a supplementary file (S3). The data regarding stillbirths were obtained and then aggregated at unit level.

Line 107 states: “outcome data was collected from routine electronic clinical data held in Trust data repositories… monthly aggregate totals”.

- the key variables for this analysis are not clearly described; for example, what were the outcomes and how were they defined/measured and at what level (individual? aggregated at higher level? repeated over time?); what was the main exposure variable and how was it defined? any covariates and how they were measured/defined for the analysis?

The protocol provided detailed information on the data sources and is publicly available and cited in the manuscript. It is clearly stated that all outcome data is aggregated to site/month, although for practical purposes some variables were aggregated by the analysis team rather than the data provider. The main exposure variable is the SBL intervention as stated in the title and the first line of the method section (lines 83-84). No covariates are mentioned as there were none, other than the Trust (line 137)

- the approach to analysis is not clearly described. Without knowing what the outcome and explanatory variables were, or the units of analysis (individual?/trust?/hospital?), it is not possible to assess whether the models described were suitable i.e. appropriate for the outcome variables, level of aggregation, any hierarchical relationships in the data, assessing change etc. For instance, the RRs in Table 2 compare post- and pre- periods (however defined), but it is not clear whether the rates in those periods are obtained from a model on the outcomes of individual women, or a model on aggregated outcomes at hospital level across 19 trusts, or even aggregated outcomes at trust level.

We argue that the analytical approach is clear in the manuscript. The main analyses are clearly stated to be based on data aggregated at the trust/month level (lines 134-135). The estimates are defined clearly as estimates 2 years before and 2 years post the nominal implementation date (Line 138) from a linear trend model (line 137). For example, the data supplied by the participating units included the number of stillbirths, number of births etc. so in some cases data were aggregated by the team.

Furthermore, the results tables should present number of units of observation contributing to the pre- and the post- rates/proportions for each outcome, not just the overall totals as currently presented. Ideally, for the reporting of outcomes, assuming they are all rates, you should report the number of units (e.g. individuals), the number of events and the rate in the pre- and in the post- period, followed by the rate ratio, confidence intervals and p-value for the test of comparison of the two periods.

Although the data analysed were obtained from individual units the relevant number is the number of women in the model (i.e. the totals that are presented), and the RR presented is based on the slope over time – converted to a pre-post RR for ease of interpretation.

Crude and adjusted rate ratios/CIs/p-values should be presented if unadjusted and adjusted models were used.

There were no adjusted models for the primary analyses (other than the inclusion of Trust). The secondary analyses by implementation status did include covariates, but as this was a secondary analysis we believe that adding unadjusted results would confuse rather than enhance the manuscript. This was done as a sensitivity analysis and made no substantive difference to the result.

For all outcomes, please be clear about how they are aggregated; for example when the change in the number of women smoking is reported as 14.3% to 11.8%, was this pooled across all women in the two periods or was this first aggregated at hospital or trust level and then pooled and compared? The answer to this question is important because it makes a difference in how the variability of the outcome is determined and whether this is indeed a statistically significant difference or not (this also applies to how the rate ratios are calculated all through).

Smoking data is (see table 3) derived from two sources. The Electronic data is aggregated on the per trust per month basis as described above. The women’s questionnaire data was analysed in the same way as the audit data (This has been clarified by adding “and women’s questionnaire data” to line 145). With specific regard to the reviewer’s comment on the change in frequency of smoking, 14.3% is the point estimate 2 years prior to the implementation of the SBL care bundle and 11.8% is at the assessment point 2 years after the implementation. These data were obtained by calculating the pooled proportion of women who smoked aggregated from the individual unit data.

Overall, I would recommend a clearer overall reporting (especially of the methods and results) to be consistent with the reporting guidelines appropriate for this study, and a more detailed description of the statistical methods to enable assessment of their suitability and potential replication by authors of similar studies

We believe all the information requested was available in brief in the manuscript and in detail in the published protocol. A few details have been clarified in the manuscript where these were missing. We have adhered to the STROBE reporting guidleines.
